# Single-Particle Detection of Transcription following Rotavirus Entry

**DOI:** 10.1128/JVI.00651-17

**Published:** 2017-08-24

**Authors:** Eric N. Salgado, Srigokul Upadhyayula, Stephen C. Harrison

**Affiliations:** a Laboratory of Molecular Medicine, Boston Children's Hospital, Harvard Medical School, Boston, Massachusetts, USA; b Howard Hughes Medical Institute, Boston, Massachusetts, USA; c Departments of Cell Biology and Pediatrics, Harvard Medical School, and Program in Cellular and Molecular Medicine, Boston Children's Hospital, Boston, Massachusetts, USA; University of Pittsburgh School of Medicine

**Keywords:** RNA synthesis, fluorescence microscopy, rhesus rotavirus, virus entry

## Abstract

Infectious rotavirus particles are triple-layered, icosahedral assemblies. The outer layer proteins, VP4 (cleaved to VP8* and VP5*) and VP7, surround a transcriptionally competent, double-layer particle (DLP), which they deliver into the cytosol. During entry of rhesus rotavirus, VP8* interacts with cell surface gangliosides, allowing engulfment into a membrane vesicle by a clathrin-independent process. Escape into the cytosol and outer-layer shedding depend on interaction of a hydrophobic surface on VP5* with the membrane bilayer and on a large-scale conformational change. We report here experiments that detect the fate of released DLPs and their efficiency in initiating RNA synthesis. By replacing the outer layer with fluorescently tagged, recombinant proteins and also tagging the DLP, we distinguished particles that have lost their outer layer and entered the cytosol (uncoated) from those still within membrane vesicles. We used fluorescent *in situ* hybridization with probes for nascent transcripts to determine how soon after uncoating transcription began and what fraction of the uncoated particles were active in initiating RNA synthesis. We detected RNA synthesis by uncoated particles as early as 15 min after adding virus. The uncoating efficiency was 20 to 50%; of the uncoated particles, about 10 to 15% synthesized detectable RNA. In the format of our experiments, about 10% of the added particles attached to the cell surface, giving an overall ratio of added particles to RNA-synthesizing particles of between 250:1 and 500:1, in good agreement with the ratio of particles to focus-forming units determined by infectivity assays. Thus, RNA synthesis by even a single, uncoated particle can initiate infection in a cell.

**IMPORTANCE** The pathways by which a virus enters a cell transform its packaged genome into an active one. Contemporary fluorescence microscopy can detect individual virus particles as they enter cells, allowing us to map their multistep entry pathways. Rotaviruses, like most viruses that lack membranes of their own, disrupt or perforate the intracellular, membrane-enclosed compartment into which they become engulfed following attachment to a cell surface, in order to gain access to the cell interior. The properties of rotavirus particles make it possible to determine molecular mechanisms for these entry steps. In the work described here, we have asked the following question: what fraction of the rotavirus particles that penetrate into the cell make new viral RNA? We find that of the cell-attached particles, between 20 and 50% ultimately penetrate, and of these, about 10% make RNA. RNA synthesis by even a single virus particle can initiate a productive infection.

## INTRODUCTION

Pathways of virus entry vary not only among viruses (1–3) but also, in many cases, according to the state and identity of the cell being infected. The conserved features, for any particular type of virus, are the molecular events in the virion itself that direct the multistep process of turning an extracellularly packaged genome into an intracellularly transcribing or replicating one. The likelihood that a given virion will succeed in initiating infection is a function of the efficiency of each of the steps in the entry pathway. Imaging of single virus particles as they enter cells, made possible by advances in fluorescence microscopy (4), offers a way to connect *in situ* observation of the entry process with virion structure and biochemistry.

**FIG 1:**
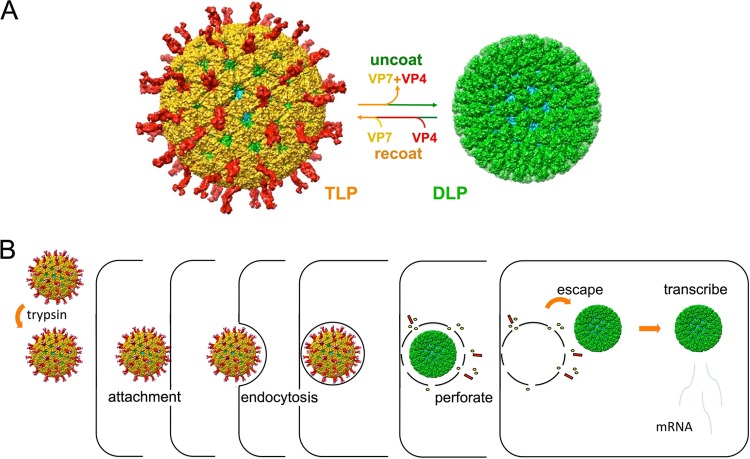
Rotavirus structure, entry pathway, and infectivity. (A) Triple-layer particle (TLP) and double-layer particle (DLP) interconversion. Outer-layer proteins VP4 (red) and VP7 (yellow) and DLP proteins VP6 (green) and VP2 (cyan) are shown. Chelation of Ca^2+^ ions dissociates VP7 trimers, stripping off the outer layer of the TLP. Adding back VP4 and VP7 in the presence of Ca^2+^ recoats the particle and restores infectivity (19). (B) Outline of RRV entry pathway (8). Trypsin activates the TLP by introducing a cleavage between VP8* (the globular tip of the VP4 spike) and VP5* (the “body” and “foot” of the spike). The particle attaches to cells by interaction of VP8* with sialylated glycolipids and endocytoses, probably by generating its own (clathrin-independent) uptake vesicle. Events, still to be determined, within the uptake vesicle lead promptly (in general, within 10 min) to loss of the outer-layer proteins and escape of the transcriptionally active DLP into the cytosol.

Initial steps in rhesus rotavirus (RRV) entry are attachment by binding with a glycan receptor (in several well-characterized cases, a glycolipid head group [5–7]) and subsequent uptake into small vesicles. The endocytic step is clathrin independent in the cells we have studied (8), although it might involve clathrin in other cell types or for other rotavirus strains. Penetration is directly from the uptake vesicles, rather than from larger endosomes (8). Figure 1A defines the components of a rotavirus particle, by reference to molecular structures obtained by electron cryomicroscopy (cryo-EM) and X-ray crystallography. Attachment and penetration are both activities of the outer layer of the infectious, triple-layered particle (TLP) (9). The result of successful penetration is release of the double-layered particle (DLP) into the cytosol, with loss of the two outer-layer proteins, VP4 and VP7 (10, 11). Loss of Ca^2+^, which stabilizes the trimeric VP7, triggers these events (11). VP4, activated by cleavage to VP8* and VP5* (12, 13), is the molecular agent of penetration (14, 15), with folding back of the VP5* trimer likely to be the essential membrane-disruptive step (16, 17).

The outer-layer proteins can be stripped away and replaced with recombinant equivalents (18, 19). The infectivity of recoated TLPs (rcTLPs), as measured by the ratio of particles to focus-forming units (P/FFU ratio), is at least as high as that of native virions (19). Fluorescent labeling of each of the components in the recoating reaction (VP4, VP7, and the DLP) allows one to follow by live-cell imaging their fates during cell entry. We showed in previous work that during infection of BSC-1 cells (a monkey kidney cell line), rcTLPs become insensitive to elution by EDTA within a few minutes of attachment (8). In those experiments, about 20 to 30% of the attached particles ultimately uncoated (i.e., lost VP4 and VP7) within 10 to 15 min of their initial addition to the culture medium. Particles that reached Rab5-labeled early endosomes never uncoated, and disabling Rab5 did not affect infectivity (8).

DLPs that have entered the cytosol by the route just described diffuse away rapidly from the surface-proximal site of penetration. They do not dissociate further once released into the cytosol. Within the DLPs, RNA-dependent RNA polymerase (VP1) and capping (VP3) activities synthesize, cap, and “export” into the cytosol 11 species of mRNA generated from each of the 11 genomic segments (20, 21). Removing VP4 and VP7 *in vitro* also activates these enzymes, and mRNA synthesis proceeds promptly if the required ribonucleoside triphosphates and Mg^2+^ are present (22, 23). We show here that RNA synthesis also begins promptly after uncoating in cells. In the absence of a suitable live-cell fluorescent probe for newly synthesized, rotavirus mRNA, we chose to use fluorescence *in situ* hybridization (FISH) (24, 25) with cells fixed at defined intervals following addition of labeled rcTLPs to determine the timing, efficiency, and intracellular localization of the earliest RNA synthesis following loss of VP4 and VP7. We found that for the RRV rcTLPs and BSC-1 cells in our experiments, about 5% of the particles that attach to the cells ultimately synthesize mRNA and that the number of transcribing particles per cell corresponds to the multiplicity of infection (MOI). The results define the entry steps responsible for observed inefficiencies during entry and indicate that a single transcribing particle is sufficient to initiate infection.

## RESULTS

### Labeled rcTLPs.

**FIG 2:**
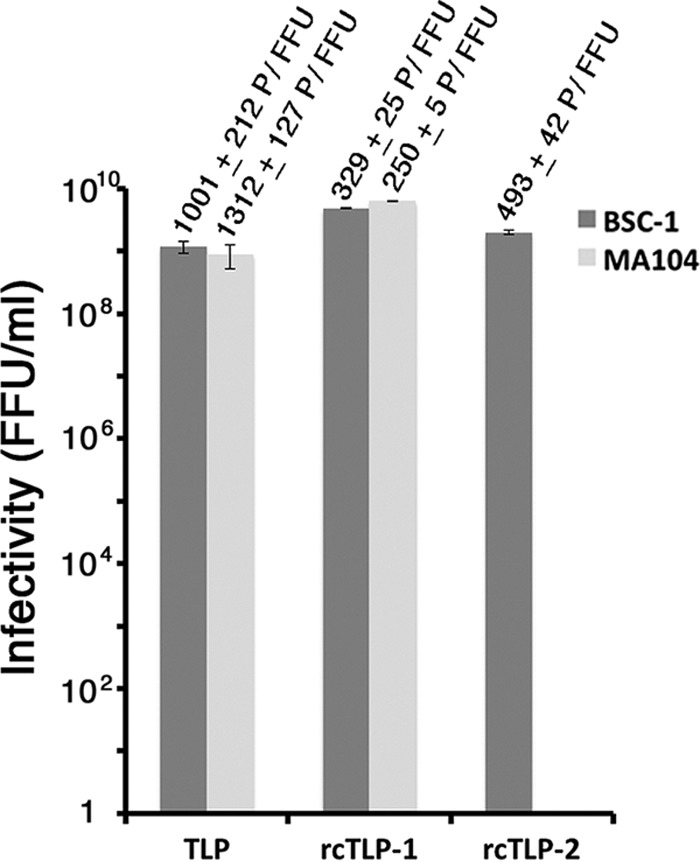
Infectivity of RRV and fluorescently labeled rcTLPs. Focus-forming assays comparing native RRV (TLP) to the two differently labeled rcTLP preparations employed in this work were performed. For rcTLP-1, the DLPs and VP7 were labeled with Atto 647N and Atto 488 dyes, respectively. For rcTLP-2, the DLPs and VP7 were labeled with Atto 488 and Atto 390 dyes, respectively. Infectivity is shown in focus-forming units per milliliter from triplicate experiments in the BSC-1 (dark gray) and MA104 (light gray) cell lines. Standard deviations of the three measurements are shown as error bars. The specific infectivity (particle per focus-forming unit [P/FFU]) of the virus in each sample is shown above the respective bars along with its standard deviation.

Optimzed labeling and recoating protocols, described Materials and Methods, yielded preparations of rcTLPs with P/FFU ratios between 250 and 500 in both BSC-1 and MA104 cells, i.e., slightly more infectious on a per-particle basis than native virions (Fig. 2).

### Atto 565-labeled oligonucleotides as probes for DLP-produced mRNA.

**FIG 3:**
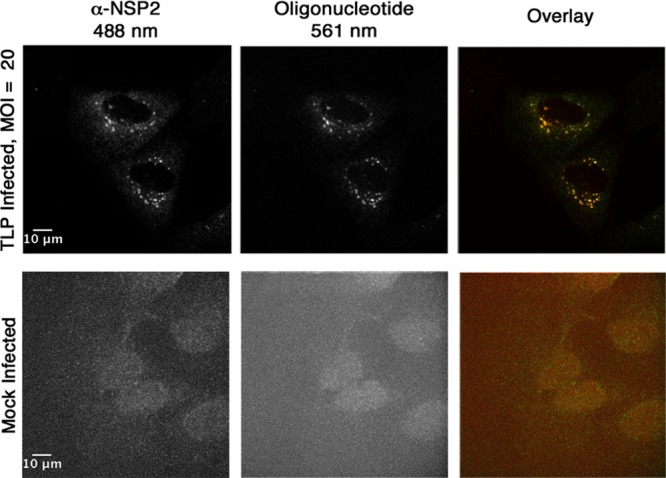
NSP2 and Atto 565 oligonucleotide probe colocalization in TLP-infected cells. BSC-1 cells were infected at an MOI of 20 (top row) or mock infected (bottom row). Infection was allowed to proceed at 37°C for 6 h. After paraformaldehyde fixation, the cells were permeabilized with 1% Triton X-100 and probed with a primary, NSP2-specific antibody, followed by incubation with a secondary IgG coupled to Alexa 488. The samples were then incubated overnight with the pool of Atto 565-labeled oligonucleotides and imaged. Maximum-intensity z-projections of the 488-nm channel (left), the 561-nm channel (middle), and the overlay of the two channels (right) are shown.

To verify that the oligonucleotides in the Atto 565-labeled probe pool could serve as markers for RRV mRNA in cells, we infected BSC-1 cells for 6 h with native, unlabeled TLPs (MOI = 20). We then fixed and probed the cells with the labeled oligonucleotides together with an antibody that recognizes RRV nonstructural protein 2 (NSP2), a major component of the “viroplasm” in which rotavirus RNA synthesis and packaging takes place (26–29). Colocalization of the probes with NSP2 in the TLP-infected cells confirmed effective probe hybridization (Fig. 3).

**FIG 4:**
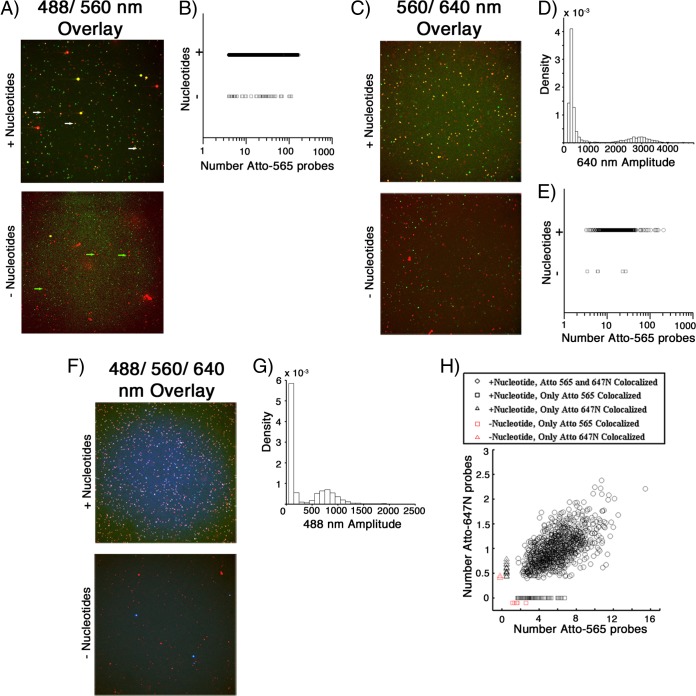
*In vitro* mRNA synthesis and labeled oligonucleotide hybridization. (A) Unlabeled DLPs were incubated with (top) or without (bottom) nucleotides to allow for mRNA production, followed by incubation with a primary VP6-specific antibody and a secondary Alexa 488-conjugated goat anti-mouse IgG. The samples were then incubated with the Atto 565 oligonucleotide probe pool and subsequently imaged in 3D. Maximum-intensity z-projections of the overlay of the 488-nm (red) and 561-nm (green) channels are shown. White arrows, DLP/oligonucleotide colocalized particles (pseudocolored yellow); green arrows, DLP signal alone (pseudocolored red). (B) Scatter plot of the number of Atto 565 oligonucleotides colocalized with Alexa 488 signal from samples with (○) and without (□) nucleotides. From samples incubated with the required nucleotides, 7,765 Alexa 488-containing particles colocalized with 4 or more Atto 565 oligonucleotide probes, with an average of ∼20 probes per particle. Conversely, only 45 particles in samples incubated without nucleotides had any significant colocalized oligonucleotide signal. (C) Atto 647N-labeled DLPs were incubated with (top) or without (bottom) nucleotides. Following incubation with the Atto 565 oligonucleotide probe pool, DLPs were imaged in 3D. Maximum-intensity z-projections of the overlay of the 640-nm (red) and 561-nm (green) channels are shown. (D) Probability density of 640-nm channel amplitudes. Detections with amplitudes less than 1,500 were not considered DLPs; detections above 4,000 were considered aggregates or multiple, unresolvable DLPs. (E) Scatter plot of the number of Atto 565 oligonucleotides colocalized with DLPs from samples with and without nucleotides. Of the 1,332 DLPs incubated with nucleotides, 1,291 (96.9%) colocalized with an average of ∼21 Atto 565 oligonucleotide probes (○), while 41 (3.1%) had no significant colocalization of probe. Of the 607 DLPs incubated without nucleotides, 602 (99.2%) had no significant colocalization of probe and 5 (0.8%) did colocalize (□). (F) Atto 488-labeled DLPs were incubated with (top) or without (bottom) nucleotides. Following incubation with both the Atto 565 and Atto 647N oligonucleotide probe pools, DLPs were imaged in 3D. Maximum-intensity z-projections of the overlay of the 488-nm (red), 561-nm (green), and 640-nm (blue) channels are shown. (G) Probability density of 488-nm channel amplitudes derived from image analysis. Detections with amplitudes of less than 400 were not considered DLPs; detections above 1,500 were considered aggregates or multiple unresolvable DLPs. (H) Scatter plot of the number of Atto 565 and Atto 647N oligonucleotides colocalized with DLPs from the samples for panel D. Of a total of 1,126 analyzed DLPs incubated with nucleotides, 1,038 (92.2%) colocalized with both probes (○), 53 (4.7%) colocalized with only the Atto 565 probe (□, black), 14 (1.2%) colocalized with only the Atto 647N probe (△, black), and 21 (1.9%) had no significant colocalization of probe. Of 152 DLPs incubated without nucleotides analyzed, 144 (94.7%) had no significant colocalization of probe, 6 (3.9%) colocalized with only the Atto 565 probe (□, red), and 2 (1.3%), colocalized with only the Atto 647N probe (△, red). Amplitude detection and quantification of the number of labeled probes were performed as described in Materials and Methods.

To ensure that mRNA produced by unlabeled and fluorescently labeled DLPs could be detected with these reagents, we incubated unlabeled DLPs or Atto 647N-labeled DLPs *in vitro* with or without substrates for RNA synthesis. After fixation, both samples were exchanged into a hybridization buffer. Prior to hybridization, unlabeled DLPs were incubated with a VP6-specific antibody and then with an Alexa 488-conjugated secondary antibody for visualization. Atto-labeled 565 probes were then added to all samples and incubated for 2 h., and the particles were subsequently imaged. With a few exceptions, only DLPs incubated with the required substrates for RNA synthesis colocalized with the fluorescent probes (Fig. 4A to E), and both unlabeled and Atto 647N-labeled DLPs colocalized with about 20 Atto 565 probes. These results show that the labeled probes can hybridize with the mRNA associated with a transcriptionally active DLP but not with the double-stranded RNA packaged within a DLP. We obtained similar results after hybridization with the Atto 565/647N dual-probe pools (Fig. 4F to H).

### FISH at low and moderate MOIs with long incubation times.

We examined mRNA production by fluorescently labeled rcTLPs at low viral loads to ensure that there were no adverse effects from overloading the cells with infectious particles. We incubated BSC-1 cells at an MOI of 1 for 5 h with doubly labeled rcTLPs containing Atto 647N-labeled DLPs, Atto 488-labeled VP7, and unlabeled VP4. We then fixed the cells, probed them with the Atto 565 oligonucleotide pool, and imaged in three dimensions with a spinning-disk confocal microscope.

**FIG 5:**
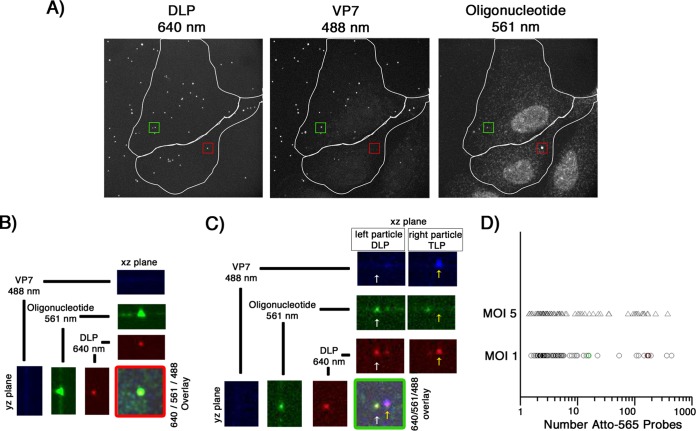
*In situ* hybridization, after long incubation times, of BSC-1 cells infected at a low MOI. (A) Cells were infected at an MOI of 1 for 10 min with doubly labeled rcTLPs and washed, and infection was allowed to continue for 5 h. Paraformaldehyde fixation was followed by overnight incubation with the pool of 44 Atto 565-labeled oligonucleotide probes and subsequent 3D imaging at 640-nm (left), 488-nm (middle), and 561-nm (right) excitation wavelengths, maximum-intensity projections of which are shown. (B) The uncoated DLP highlighted in the red box in panel A, displaying the individual channels in the *xz* and *yz* planes through the particle, as well as the maximum projection of the overlay of all three channels. (C) The uncoated DLP highlighted in the green box in panel A (white arrow), displaying the overlay of all three channels and the individual channels in the *xz* and *yz* planes through the center of the particle. For comparison, the *yz* plane of a nearby particle that has retained its VP7 shell (yellow arrow) is also shown. (D) Scatter plot of the number of Atto 565 probes colocalized with a given uncoated DLP after 5 h of infection at an MOI of 1 (○) or 5 (△). The uncoated DLPs highlighted in panels B and C are represented here as red and green circles, respectively, plotted within the data collected from cells infected at an MOI of 1. Quantification of the number of colocalized Atto 565-labeled probes was performed as described in Materials and Methods.

At this viral load, a subpopulation of uncoated DLPs (i.e., spots of fluorescence at 647 nm that lacked a detectable signal in the 488-nm channel) colocalized with strong fluorescence in the 560-nm channel (Fig. 5A to C). The number of colocalized probes, determined by comparison with the single-molecule Atto 565 amplitude (see Materials and Methods), ranged from about 1.4 to about 430, signifying detection of as few as one or two transcripts or as many as 10 copies of the entire, 11-segment genome (Fig. 5D). Only about 21% of all of the uncoated particles appeared to be generating mRNA (Table 1), even though nearly half of the particles that had attached to the cells had lost detectable VP7.

**TABLE 1:** Results for cells infected for 5 h at MOIs of 1 and 5 and probed with the Atto 565 oligonucleotide pool

MOI	Total no. of:			DLPs				
	Images	Cells	Particles bound	Uncoated		Colocalized with Atto 565 probe		
				Total no.	%	Total no.	%	Avg per cell
1	25	63	758	401	52.9	85	21.2	1.3
5	15	34	2,186	667	30.5	85	12.7	2.5

**FIG 6:**
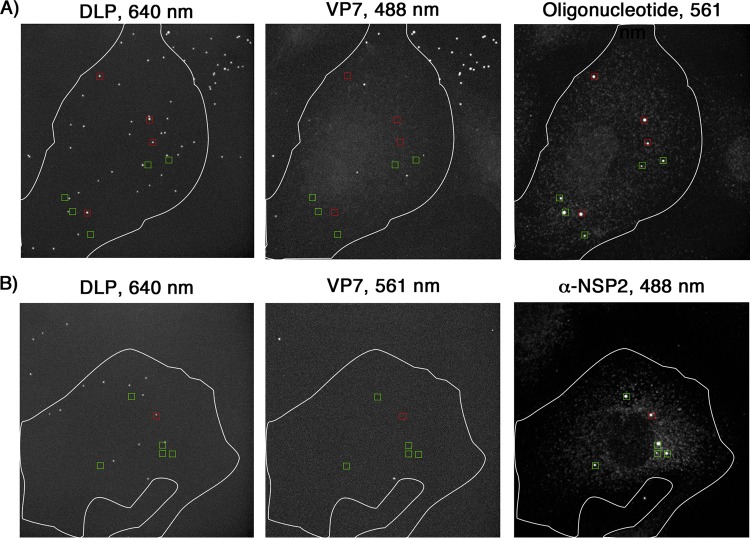
Viroplasm localization with respect to fluorescently labeled DLPs. (A) Cells were infected at an MOI of 5 for 10 min with doubly labeled rcTLPs (VP7, Atto 488; DLP, Atto 647N) and washed, and infection was allowed to continue for 5 h. Paraformaldehyde fixation was followed by overnight incubation with the pool of 44 Atto 565-labeled oligonucleotide probes and subsequent 3D imaging at 640-nm (left), 488-nm (middle), and 561-nm (right) excitation wavelengths. Maximum-intensity projections of all three channels are shown. Red boxes, uncoated DLPs (640-nm channel), as indicated by the lack of VP7 (488-nm channel), colocalized with a strong signal in the Atto 565 oligonucleotide channel (561 nm). Green boxes, similarly large oligonucleotide bodies that do not colocalize with any DLP signal. (B) Cells were infected at an MOI of 1 for 10 min with doubly labeled rcTLPs (VP7, Atto 565; DLP, Atto 647N) and washed, and infection was allowed to continue for 6 h. Paraformaldehyde fixation was followed by incubation with a primary antibody that recognizes NSP2, followed by incubation with a secondary IgG coupled to Alexa 488. The samples were then imaged at 640-nm (left), 561-nm (middle), and 488-nm (right) excitation wavelengths. Maximum-intensity projections of all three channels are shown. Red boxes, uncoated DLPs (640-nm channel), as indicated by the lack of VP7 (561-nm channel), colocalized with a strong signal in the 488-nm channel, corresponding to the presence of NSP2. Green boxes, similarly large NSP2 clusters that do not colocalize with DLP signal.

Increasing the viral load to an MOI of 5 did not increase the fraction of uncoated particles colocalizing with fluorescent probes, but it did increase the fraction of DLPs that colocalized with 10 or more (Fig. 5D; Table 1). When these samples were compared qualitatively with those infected at an MOI of 1, they appeared to have a greater number of large, oligonucleotide-containing bodies that did not colocalize with any DLP signal (Fig. 6A). We did not include these noncolocalized structures when determining the apparent transcriptional activity of uncoated DLPs. Cells infected for 6 h at an MOI of 1 with doubly labeled rcTLPs containing Atto 647N-labeled DLPs, Atto 565-labeled VP7, and unlabeled VP4, and subsequently probed with an antibody that recognizes NSP2, showed the presence of NSP2-containing viroplasms that also did not colocalize with any DLP signal (Fig. 6B).

### FISH at a high MOIs with short incubation times.

To determine how soon after uncoating we could detect nascent mRNA, we infected cells at an MOI of 20 and fixed them at 15, 30, and 60 min postinfection, choosing the time points to fall between our previously reported average time to cytosolic release of DLPs (10 min [8]) and the 1-h incubation time commonly used in rotavirus infectivity assays.

**FIG 7:**
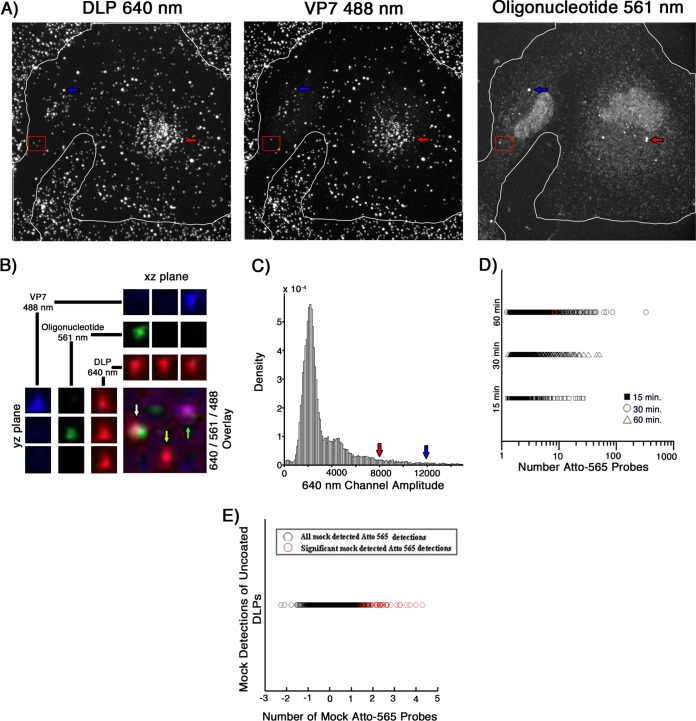
*In situ* hybridization, after short incubation times, of BSC-1 cells infected at a high MOI. (A) Cells were infected at an MOI of 20 for 15, 30, or 60 min with doubly labeled rcTLPs. Paraformaldehyde fixation was followed by overnight incubation with the pool of 44 Atto 565-labeled oligonucleotide probes and subsequent 3D imaging at 640-nm (left), 488-nm (middle), and 561-nm (right) excitation wavelengths, maximum-intensity projections of which are shown for a sample after a 60-min incubation. (B) Three fluorescently labeled particles in the red box in panel A, displaying the individual channels in the *xz* and *yz* planes through the center of each particle, as well as the maximum projection of the overlay of all three channels. White arrow, an uncoated DLP that colocalizes with ∼12 Atto 565-labeled oligonucleotides. Yellow arrow, an uncoated DLP with no colocalized oligonucleotide signal. Green arrow, an rcTLP. (C) Probability density of 640-nm channel amplitudes derived from image analysis as described in Materials and Methods. Detections with amplitudes of less than 900 were not considered a DLP, while detections above 4,000 were considered aggregates or multiple unresolvable DLPs. Red and blue arrows point roughly to the 640-nm amplitude of the particles in panel A highlighted by corresponding red and blue arrows. Particles such as these were excluded from analysis, as they likely represent aggregates or spatially unresolved particles. (D) Scatter plot of the number of Atto 565 probes colocalized with a given uncoated DLP after 15 (□), 30 (△), or 60 (○) min of infection at an MOI of 20. The particle highlighted in panels A and B is represented here as a red circle plotted within the 60-min data. (E) In images collected from mock-infected cells probed with the Atto 565-labeled oligonucleotide pool, 100 random “DLP” locations were generated, matching the average number of uncoated DLPs calculated from the data in Table 2. Of the 4,600 mock particles (black circles), 36 (red circles) colocalized with significant signal in the 560-nm channel as described in Materials and Methods. Quantification of the number of colocalized Atto 565 labeled probes was performed as described in Materials and Methods.

We found that uncoated DLPs colocalized with the mRNA probe signal even as early as 15 mins postinfection (Fig. 7; Table 2). Although the percentage of bound particles that had uncoated increased between 15 and 30 min, the percentage of these DLPs that colocalized with the oligonucleotide probes (about 6 to 8%) did not. By 60 min, 32% of the bound particles had uncoated, of which about 9% colocalized with the probe. At all time points, the average number of colocalized probes was about 4, but the population of DLPs that colocalized with greater than this average number grew over time (Fig. 7D). In contrast, random assignment of DLP locations in mock-infected samples probed with the Atto 565 pool showed only 0.8% of these mock “particles” colocalizing with a significant 560-nm amplitude at an average of about 2 probes (Fig. 7E).

**TABLE 2:**
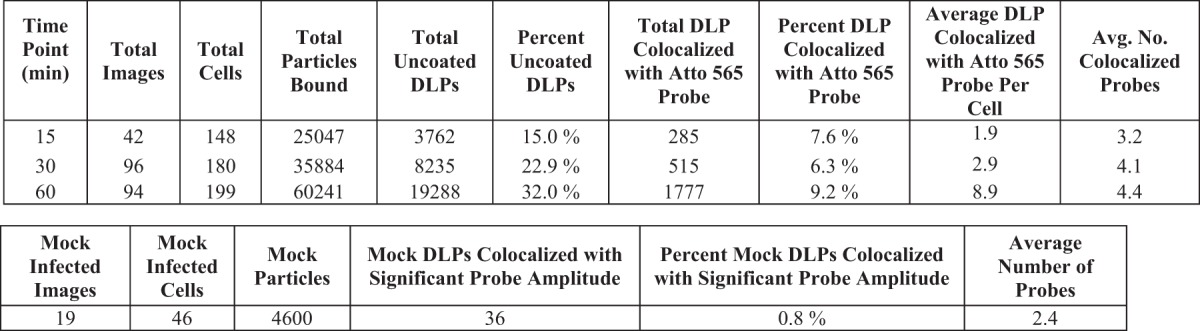
Results for cells infected at an MOI of 20 for 15, 30, and 60 min, or mock infected, and probed with the Atto 565 oligonucleotide pool

For further validation of these results, we probed cells infected at an MOI of 5 with a dually labeled oligonucleotide pool. We split the pool of 44 oligonucleotides in two, with two probes for each of the 11 rotavirus genes labeled with Atto 565 or Atto 647N, respectively. We used both probe pools to examine BSC-1 cells infected with rcTLPs for 15, 30, or 60 min. For this purpose, we labeled DLPs with Atto 488 and VP7 with Atto 391 dye, thus allowing four-color fluorescence acquisition.

**FIG 8:**
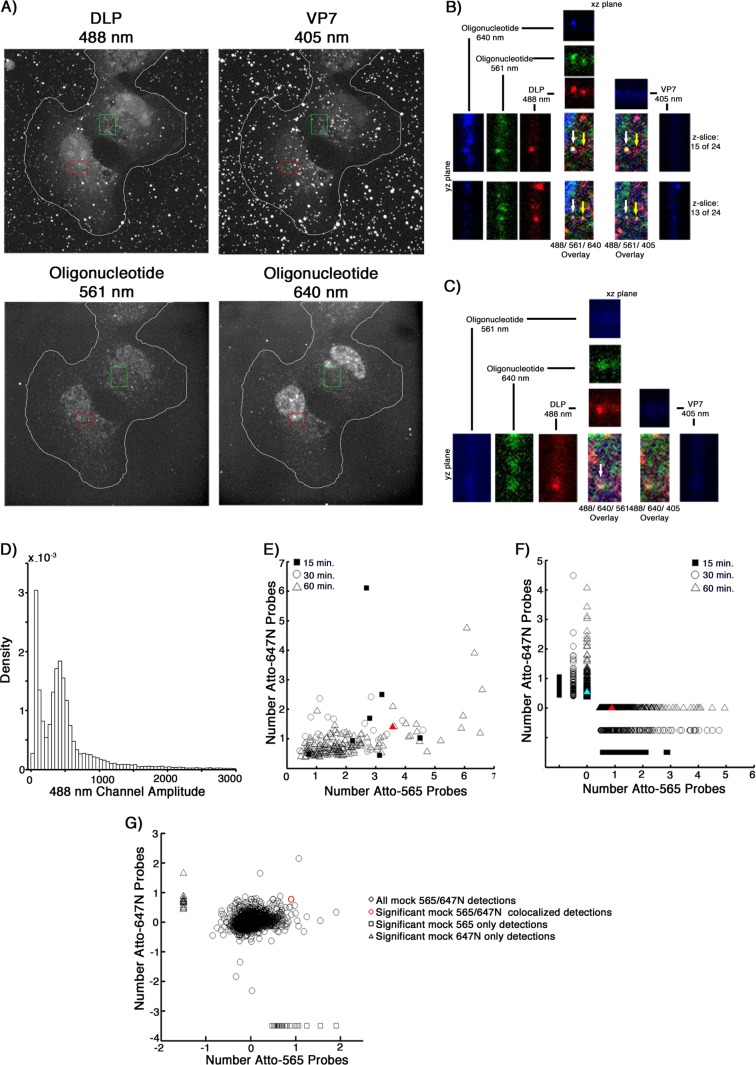
*In situ* hybridization with dual-probe oligonucleotides of BSC-1 cells infected at an MOI of 5. (A) Cells were infected at an MOI of 5 for 15, 30, or 60 min with doubly labeled rcTLPs. Paraformaldehyde fixation was followed by overnight incubation with two pools of 22 Atto 565- or Atto 647N-labeled oligonucleotide probes and subsequent 3D imaging at 488-nm (top left), 405-nm (top right), 561-nm (bottom left), and 640-nm (bottom right) excitation wavelengths, maximum-intensity projections of which are shown for a sample after a 60-min incubation. (B) Uncoated DLPs highlighted in the red box in panel A, displaying the individual channels in the *xz* and *yz* planes through a DLP colocalized with both probes (white arrow) or with only the Atto 565 probe (yellow arrow), as well as the maximum projection of the overlay of three channels. (C) An uncoated DLP highlighted in the green box in panel A that colocalizes only to the Atto 647N probes (white arrow), displaying the individual channels in the *xz* and *yz* planes through the center of the particle, as well as the maximum projection of the overlay of three channels. (D) Probability density of 488-nm channel amplitudes derived from image analysis as described in Materials and Methods. Detections with amplitudes of less than 200 were not considered a DLP, while detections above 750 were considered aggregates or multiple unresolved DLPs. (E) Scatter plot of colocalized Atto 565 and Atto 647N probes derived from DLPs that colocalize with a signal in both channels. The number of each respective probe colocalized with a given uncoated DLP after 15 (■), 30 (○), or 60 (△) min is shown as a scatter plot. The DLP highlighted with the white arrow in panel B is plotted as a red triangle. (F) Scatter plot of colocalized Atto 565 or Atto 647N probes derived from DLPs that colocalize with one or the other probe. The number of respective probes after 15 (■), 30 (○), or 60 (△) min is shown. The DLP highlighted by the yellow arrow in panel B is shown as a red triangle, and the DLP highlighted in panel C is shown as a light blue triangle. (G) In images collected from mock-infected cells probed with combined Atto 565/Atto 647N-labeled oligonucleotide pools, 40 random “DLP” locations were generated, matching the average number of uncoated DLPs calculated from the data in Table 3. Of the 2,400 mock particles (○, black), only 1 (○, red) colocalized with a significant signal in both the 560-nm and the 640-nm channels, 27 (□) colocalized only with a significant 560-nm signal, and 19 (△) colocalized only with a significant 640-nm signal. Quantification of the number of colocalized Atto 565- or Atto 647N-labeled probes was performed as described in Materials and Methods.

We found four different populations of uncoated DLPs (Fig. 8): those with no colocalized oligonucleotide signal, those colocalized with either a 561- or a 640-nm signal (Fig. 8B, C, and F), and those colocalized with signals in both channels (Fig. 8B and E). From 15 to 60 min, the percentage of uncoated particles that colocalized with both probes stayed roughly the same (∼1%, with an average of ∼2 of each probe) (Table 3). The percentage of total colocalized DLPs during the 60-min time period increased from about 10% after 15 min to about 13% after 60 min, yielding values similar to those determined with the single probe pool. Random DLP position assignments generated from mock-infected, dual-oligonucleotide-probed samples showed a probe signal at 2% of the positions, with an average of 0.8 Atto 565 or 0.7 Atto 647N probes (Fig. 8G); only a single one of these detections corresponded to a random colocalization of both probes out of 2,400 mock positions chosen.

**TABLE 3:**
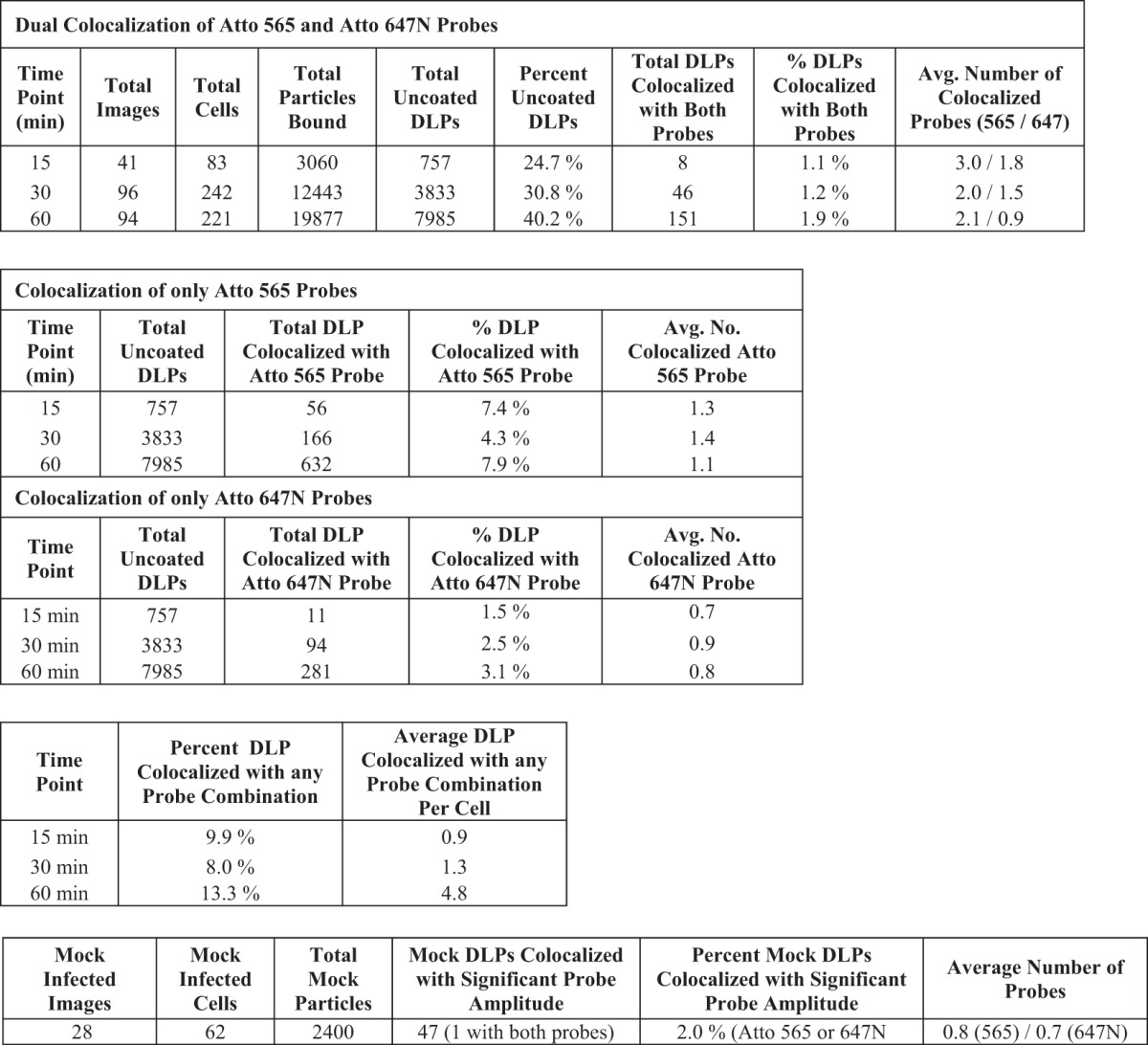
Results for cells infected at an MOI of 5 for 15, 30, and 60 min, or mock infected, and probed with the 22 oligonucleotide pools labeled with Atto 565 or Atto 647N

We also carried out similar experiments with MA104 cells, the line commonly used for maintaining native rotavirus stocks. These cells derive from the same host (Cercopithecus aethiops) as do BSC-1 cells. The infectivity of fluorescently labeled rcTLPs was the same in both cell lines and comparable to that of purified, unlabeled native virions (Fig. 2).

**FIG 9:**
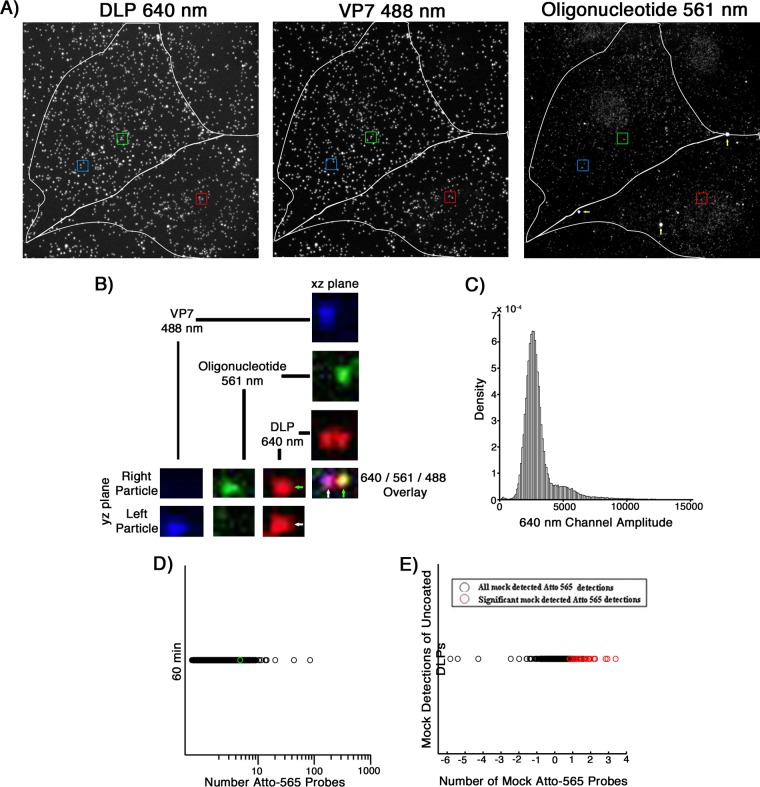
*In situ* hybridization after 60 min of infection of MA104 cells at an MOI of 20. (A) Cells were infected at an MOI of 20 for 60 min with doubly labeled rcTLPs. Paraformaldehyde fixation was followed by overnight incubation with the pool of 44 Atto 565-labeled oligonucleotide probes and subsequent 3D imaging at 640-nm (left), 488-nm (middle), and 561-nm (right) excitation wavelengths, representative maximum-intensity projections of which are shown. Red, green, and blue boxes, DLPs colocalized with ∼4 Atto 565-labeled oligonucleotides. White arrows, likely oligonucleotide aggregates. (B) Fluorescently labeled particles in the green box in panel A, displaying the individual channels in the *xz* and *yz* planes passing through the center of each particle, as well as the maximum projection of the overlay of all three channels. Green arrow, an uncoated DLP that colocalizes with ∼4 Atto 565-labeled oligonucleotides. White arrow, an intact fluorescently labeled rcTLP with no colocalized oligonucleotide signal. (C) Probability density of 640-nm channel amplitudes derived from image analysis as described in Materials and Methods. Detections with amplitudes of less than 900 were not considered DLPs; detections above 4,000 were considered aggregates or multiple unresolvable DLPs. (D) Scatter plot of the number of Atto 565 probes colocalized with a given uncoated DLP after 60 min of infection at an MOI of 20. The particle highlighted in the green box in panel A and shown in B is represented here as a green circle plotted within the data. (E) In images collected from mock-infected cells probed with the Atto 565-labeled oligonucleotide pool, 30 random “DLP” locations were generated, matching the average number of uncoated DLPs calculated from the data in Table 3. Of the 2,460 mock particles (black circles), 28 (red circles) colocalized with significant signal in the 560-nm channel as described in Materials and Methods. Quantification of the number of colocalized Atto 565-labeled probes was performed as described in Materials and Methods.

Confluent MA104 monolayers were infected at an MOI of 20 for 1 h before fixation and probing with the Atto 565 oligonucleotide pool (Fig. 9; Table 4). From these data, 16% of the bound particles had uncoated, of which ∼32% colocalized with RNA probes (Fig. 9D). Random assignment of DLP locations in mock-infected samples similarly probed showed only 1.1% of these mock “particles” with significant 560-nm amplitudes (Fig. 9E).

## DISCUSSION

We summarize our observations as follows. (i) As we have shown previously (8), we can reconstitute infectious rotavirus particles from DLPs and outer-layer proteins after labeling each independently with a chosen fluorophore. The labeling, when carried out with the protocol described, has no effect on the infectivity of the recoated DLPs. (ii) We can detect, by fluorescent *in situ* hybridization, nascent mRNA produced by the fluorophore-labeled DLPs after entry and uncoating of the rcTLPs in cells. (iii) Of the 20 to 50% of the attached particles that ultimately uncoat, about 10 to 15% colocalize with the RNA probes we prepared. (iv) These results are similar for RRV in two different, primate-derived cell lines. (v) Accumulation of viral mRNA transcripts and NSP2-containing viroplasms can occur in locations distant from transcribing DLPs.

**FIG 10:**
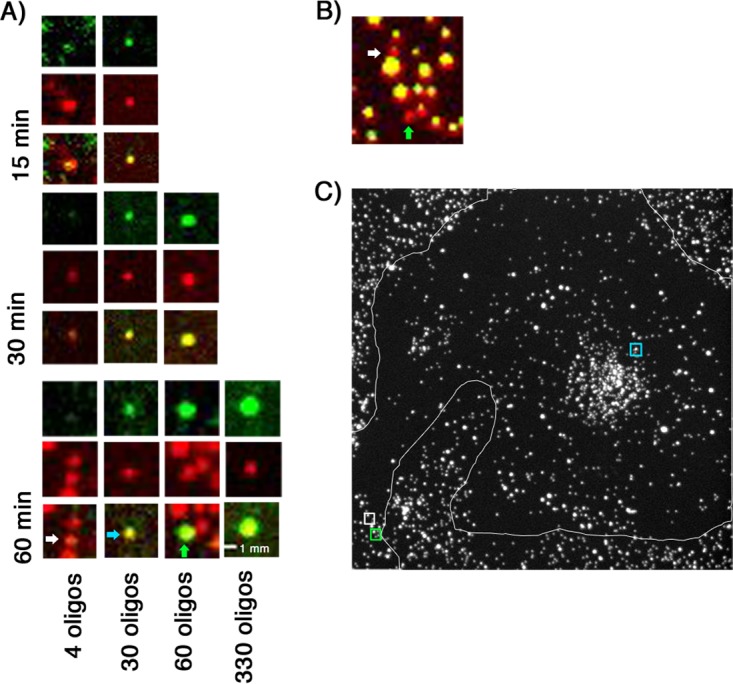
Accumulation of mRNA in doubly labeled rcTLP-infected cells. (A) Atto 647N-labeled DLPs (red) colocalize with various numbers of Atto 565-labeled oligonucleotides (green) taken from images after 15, 30, and 60 min of incubation with doubly labeled rcTLPs. Particles of interest are arranged vertically as follows: top, 560-nm channel; middle, 640-nm channel; and bottom, overlay. The numbers of colocalized oligonucleotides increase from left to right, with the rightmost particle representing the maximum number of colocalized oligonucleotides from all images collected at the respective time point. The white, blue, and green arrows point to the particles of interest from a single image collected after 1 h of incubation. (B) z-projection of the region surrounding the 4 and 60 oligonucleotide-colocalized particles from the 1-h data in panel A. Red represents the labeled DLP as in panel A, and yellow represents a particle that colocalizes with Atto 488-labeled VP7 and so has yet to lose the VP7 shell. White and green arrows correspond to the particles of interest as in panel A. (C) z-projection of the 640-nm channel of a 1-h-infected cell. The representative 4, 30, and 60 oligonucleotide-colocalized DLPs were taken from this image, with their respective locations in the cell marked by colored boxes (white, green, and blue), corresponding to the arrows in panel A.

The time course experiments at higher MOIs (Fig. 7 and 8; Tables 2 and 3) detected transcriptionally active particles within 15 min of adding the rcTLPs to cells. Even after an hour, however, transcriptionally active particles had not accumulated at any fixed location in the cell, and morphologically identifiable viroplasms were not yet present. We found transcribing particles at the cell periphery, near other particles still coated with VP7, as well as closer to the nucleus (Fig. 10). At the longer times, a few of the transcribing particles had accumulated RNA corresponding to more than one genome: in the example in Fig. 10, the distribution of DLP-associated RNA had reached or exceeded the diffraction limit. We obtained similar results with MA104 cells infected for 60 min: although the uncoating efficiency was somewhat lower, the fraction of uncoated particles yielding detectable transcripts was correspondingly higher.

A rapid sequence of penetration, uncoating, and RNA synthesis, following initial attachment, is consistent with our previous live-cell imaging observations, which showed prompt engulfment of attached particles, apparently into small, vesicular structures, from which the DLPs penetrated (8). Although some particles reached Rab5-positive endosomes, none of those particles uncoated. Uncoating *in vitro* leads immediately to RNA synthesis if substrate nucleotides are present (Fig. 4), and we expect the same to be true when DLPs enter the cytosol.

The ratio of particles to focus-forming units (FFU) for our recoated rotavirus preparations is between 250 and 500 (Fig. 2). In our experiments at an MOI of 1 (i.e., 250 to 500 particles added to the medium per cell), we detected an average of 12 rcTLPs bound per cell after 5 h, or an attachment efficiency under our conditions of about 3 to 5% (Table 1). Of these ∼12 attached particles, ∼6 ultimately uncoated, of which one (on average) generated detectable mRNA. Similar estimates for the experiments at higher MOIs yielded about 11 transcribing particles per cell for an MOI of 20 (Table 2), 4 per cell for an MOI of 5 (Table 3), and 10 per cell for an MOI of 20 in MA104 cells (Table 4). We infer from the reasonable agreement of yield, in all three cases, with multiplicity of infection that the probes probably reported most of the transcriptionally active particles. Moreover, within the uncertainty of these estimates, even a single transcribing particle may have been adequate for productive infection.

**TABLE 4:**
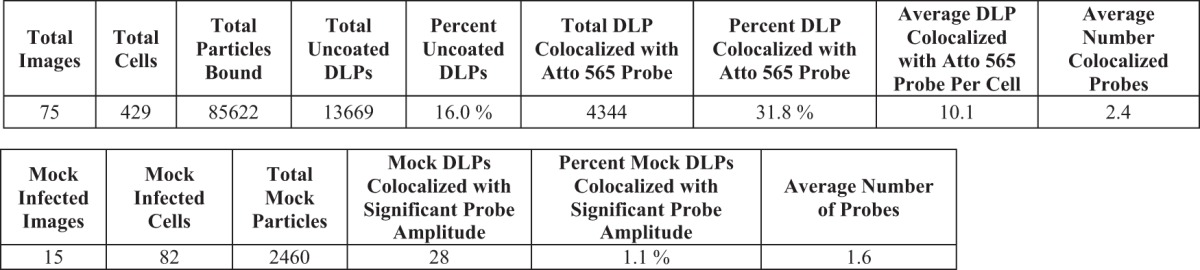
Results for MA104 cells infected at an MOI of 20 for 60 min, or mock infected, and probed with the Atto 565 oligonucleotide pool

Several mechanisms might account for the failure of many uncoated particles to generate detectable transcription products. First, some might have synthesized RNA, but at a level not detected by the FISH assay. The agreement (within a factor of about 2) between MOI and yield of particles that generated detectable RNA suggests that the fraction of unscored particles is probably modest. Second, some of the nontranscribing particles might still have been surrounded by vesicle membrane. We have interpreted the onset of rapid diffusional motion of the DLP as release from a disrupted membrane-enclosed compartment (8). The kinetics of decline in VP7 signal reported previously suggest that VP7 release is often gradual, however, and that a variable delay may intervene between the time of complete loss of VP7 and the time at which the DLP starts to diffuse rapidly in the cytosol. Moreover, we do not yet know what retains the engulfed particle in the vicinity of the cell surface prior to onset of the decline in VP7 (and VP4) signal and therefore what must happen (in addition to vesicle disruption) to allow the DLP to escape surface-proximal confinement. Third, even if fully released from the vesicle, some DLPs might retain VP7 at levels below the fluorescence detection threshold but sufficient to block transcription (e.g., by occluding the RNA exit channel or by preventing the conformational change in the VP2 shell that accompanies complete uncoating [10]). Fourth, one or more cellular mechanisms (e.g., RNA degradation) might (stochastically) limit transcript accumulation. Fifth, gradients of nucleoside triphosphates in the cell could make certain positions in the cell more hospitable than others to rapid transcript initiation and elongation. Whatever the explanation for the yield of this final step under the cell culture conditions in our experiments, an overall ratio of about 1:10 for transcribing to uncoated particles indicates both a relatively efficient chain of intracellular events and a relatively accurate RNA packaging mechanism, ensuring an infectious outcome from one or a very few entering particles.

## MATERIALS AND METHODS

### Cells.

Adherent Cercopithecus aethiops BSC-1 and MA104 cells (ATCC) were maintained at 37°C and 5% CO_2_. BSC-1 cells were grown in Dulbecco modified Eagle medium (DMEM) (Invitrogen Corporation) supplemented with 10% fetal bovine serum (FBS) and 1× GlutaMAX (Thermo Fisher Scientific). MA104 cells were similarly maintained in M199 with 10% FBS.

### TLP, DLP, and recombinant protein purification.

TLPs, DLPs, VP7, and VP4 were purified as previously described (11). For TLP and DLP production, MA104 cells were grown in 850-cm^2^ roller bottles (Corning), and confluent monolayers were infected with rhesus rotavirus (RRV) (G3 serotype) at an MOI of 0.1 focus-forming unit (FFU)/cell in M199 medium supplemented with 1 mg/ml porcine pancreatic trypsin (Worthington Biochemical). Cell culture medium was collected at 24 to 36 h postinfection, when cell adherence was <5%. TLPs and DLPs were purified by freeze-thawing, ultracentrifugation, Freon-113 extraction, and separation on a cesium chloride gradient. TLPs were desalted with a 5-ml Zeba spin column (Thermo Fisher) into 20 mM Tris pH 8.0, 100 mM NaCl, and 1 mM CaCl_2_ (TNC). DLPs were desalted into 20 mM HEPES (pH 7.5)–100 mM NaCl (HN). Infectivities of native virions and recoated rcTLPs were determined as previously described (19, 30).

VP7 and VP4 were expressed in Sf9 cells infected with a baculovirus vector. VP7 was purified by successive affinity chromatography on concanavalin A and monoclonal antibody (MAb) 159, specific for the VP7 trimer (elution was with EDTA). Purified VP7 was desalted into 2 mM HEPES (pH 7.5)–10 mM NaCl–0.1 mM CaCl_2_ (0.1 HNC). For VP4, harvested cells were lysed by freeze-thawing and clarified by centrifugation after addition of cOmplete EDTA-free protease inhibitor (Roche). VP4 was precipitated by addition of ammonium sulfate to 30% saturation, pelleted, and resuspended in 25 mM Tris (pH 8.0)–10 mM NaCl–1 mM EDTA, which was matched to the conductance of the Phenyl HP start buffer (25 mM Tris [pH 8.0] 3.5 M NaCl, 1 mM EDTA), and the solution was loaded onto a Phenyl HP column (GE Healthcare). Following elution with 25 mM Tris (pH 8.0)–10 mM NaCl–1 mM EDTA, fractions containing VP4 were pooled, dialyzed against the same buffer, loaded onto a HiTrap Q column (GE Healthcare), and eluted in Phenyl HP start buffer. Pooled fractions containing VP4 were then concentrated to 600 μl with a Centriprep 50 concentrator (Millipore) and subjected to a final purification on an S200 size exclusion column (GE Healthcare) in 20 mM HEPES (pH 7.5)–100 mM NaCl–1 mM EDTA (HNE).

### Fluorescent labeling of DLPs and VP7.

Fifty micrograms of DLPs was brought to a volume of 100 μl in HN, to which was added 11.1 μl 1 M NaHCO_3_, pH 8.3. This solution was then added to 1.11 μl of 500 μg/ml Atto 647N or 582 μg/ml Atto 488 *N*-hydroxysuccinimide (NHS) ester dye. The reaction proceeded for 1 h at room temperature before quenching with 12 μl of 1 M Tris, pH 8.0. The sample was then desalted through a 0.5-ml Zeba spin column into 20 mM Tris (pH 8.0)–100 mM NaCl.

VP7 was brought to 1.08 mg/ml in a total volume of 75.5 μl using 0.1 HNC, and 8.4 μl of 1 M NaHCO_3_ (pH 8.3) was added. This solution was mixed into 0.84 μl of 340 μg/ml Atto 488, 760 μg/ml Atto 565, or 150 μg/ml Atto 390 NHS ester dye. The reaction proceeded at room temperature for 1 h before quenching with 9 μl of 1 M Tris, pH 8.0. The labeled VP7 was then desalted into 2 mM Tris (pH 8.0)–10 mM NaCl–0.1 mM CaCl_2_ (0.1 TNC).

### Preparation of doubly labeled rcTLPs.

Recoating followed previously described protocols (19) using the labeled VP7 and DLP prepared as outlined above, along with unlabeled VP4. Briefly, 1 M sodium acetate (pH 5.2) was added to a volume of Atto-labeled DLPs resulting in a final concentration of 100 mM sodium acetate. VP4 was then added to a final concentration of 0.9 mg/ml (∼33-fold excess), and the mixture was incubated at room temperature for 1 h. Labeled VP7 was then added in a 2.3-fold excess along with a final addition of sodium acetate to maintain its concentration at 100 mM and CaCl_2_ to reach a Ca^2+^ concentration of 1 mM. The mixture was incubated at room temperature for 1 h. Recoated particles were separated from excess labeled components by cesium chloride gradient centrifugation, desalted with a 5-ml Zeba spin column into 20 mM Tris (pH 8.0), 100 mM NaCl, and 1 mM CaCl_2_ (TNC), and concentrated to about 75 μl with a 100-kDa-cutoff Microcon filter (Millipore). Titers of recoating reaction products were determined by infectious focus assays as previously described (19, 30).

### Labeled oligonucleotide probes.

Eleven sets of 4 oligonucleotide probes were generated for use in the *in situ* hybridization experiments. The four in each set were 20 bases in length and complementary to one of the 11 mRNA sequences of the rotavirus genome (GenBank accession numbers EU636924 to EU636934). The probe sequences were generated using the Stellaris RNA FISH probe designer (Biosearch Technologies, Inc., Petaluma, CA), ignoring the first 21 nucleotides to avoid sequence similarities in the 5′ untranslated region of each gene (31). Integrated DNA Technologies (IDT) synthesized the 44 total oligonucleotides with a 5′ primary amino functional group attached through a six-carbon linker. The sequences of all 44 probes are listed in Table 5.

**TABLE 5:** Sequences of the 44 20-base oligonucleotides generated for the 11 RRV gene segments using the Stellaris RNA FISH probe designer^*a*^

Gene product (gene accession no.)	Probe sequences
VP1 (EU636924)	AGATTAGATTATACTTCCCC, TGAACTGCGGATTGTGAGTT, AGCTTTTTCAATGATAGTCC, GGCATTCTCTATAACATCGC
VP2 (EU636925)	TAAATTCGTCTCACGACGCG, CTTCTTTTTCTTGCATTCGA, TGAGATAGCACTTTCTCTGA, TATCTGTAATCACCTCTTCC
VP3 (EU636926)	ATGTTTACACCATTAGAGGT, CACACCGTGTCTTAAAGCTA, ATAGATTTGGGTGTCTGCAT, TGCATTCTCGTAACTGTCTT
VP4 (EU636927)	TCATCAGATAAGTCAACGGT, TTTGCGTTTTTGTAGATCCA, TGAGCAAAAGGACCTGGATT, AGTTAACTGGAGCATAACCT
NSP1 (EU636928)	AAAGGTTGCCATGGCTAACA, TTATTCGAAGAGACTGGAGT, CTAGCGAACATCCGTGACAA, TGGTTCATTGTCCAAGAAGC
VP6 (EU636929)	ACAAGGAGTACAGGACATCC, TGGAGTATAATGTGCCTTCG, CCAATTCCTCCAGTTTGAAA, TCCAATTTCTAATCGGCAGA
NSP3 (EU636930)	ACTCCATCTTGAGCATCAAC, AGAAGAAGCCATCTGCTGAG, ACTGCAGCTTCAAATGAAGA, CAAGAGTAGAAGTTGCAGCA
NSP2 (EU636931)	TACACCGCAAGGCTCAAACG, CAAAAGCAAGCTAGCTCAGC, CTATAGCTATCGTTCTCCAA, GGTGCGATACCATAAATTAT
VP7 (EU636932)	AAAAGGAGCTAACCGCTAGC, TGAGCGATATCAGAAAGGTT, GTCCATCATTCTAGTCAAAG, GGGCTTTTAGTAATGGTGAC
NSP4 (EU636933)	AAGCTTTTCCATCTTTCCGC, CTCAATGTGTAGTTGAGGTC, TATGGAAAATACGCCATTCC, GTACAGTTAGGACAGAAGCA
NSP5 (EU636934)	GTCACGTCAATACTGAGAGA, TGCTGGAGGAAATAGATGGA, CCAGAAAGAGTTGACGTCGT, GTTCACTCCTACCAATAGAT

a Biosearch Technologies, Inc., Petaluma, CA.

To label the RNA probes with Atto dyes, 5-μl portions of each of the probes, at 100 μM in water, were pooled, and NaHCO_3_ (pH 8.3) was added from a 1 M stock a final concentration of 90 mM,. The buffered probes were then added to 15.6 μl of 10 μg/ml Atto 647N NHS ester or 18.5 μl of 10 μg/ml Atto 565 NHS ester at approximately a 1:10 molar ratio of probe to dye. The labeling reaction was allowed to proceed at room temperature for 6 h, after which 3 M NaCl was added to a final concentration of 90 mM; 640 μl of 190 proof ethanol (EtOH) was then added, and the samples were incubated on ice for 30 min. The probes were then pelleted at 4°C for 30 min at 12,000 × *g* and washed twice with 600 μl of 70% EtOH. The pellet was finally resuspended in 0.1 M triethylammonium acetate (TAA).

Labeled probes were purified by high-pressure liquid chromatography (HPLC) through a Resource RPC reverse-phase column (GE Healthcare Life Sciences) equilibrated in 0.1 M TAA and eluted with acetonitrile. Peaks with absorbance at both 260 nm and the wavelength corresponding to the appropriate label were pooled and lyophilized overnight. Lyophilized oligonucleotides were then resuspended in RNase-free water, aliquoted, and frozen at −20°C.

### *In vitro* mRNA synthesis by fluorophore-labeled DLPs.

Atto-labeled DLPs (2.5 μg) were added to a total volume of 50 μl containing 100 mM HEPES (pH 7.5), 150 mM NaCl, 9 mM MgCl_2_, 0.68 mM *S*-adenosylmethionine (NEB), and 2,000 U/ml murine RNase inhibitor (NEB), with and without 4 mM ATP and 2 mM CTP, GTP, and UTP. Samples were incubated at 37°C for 2 to 5 min, after which 5.6 μl of formalin was added and incubated at room temperature for 10 min before quenching with 6.2 μl of 1 M Tris, pH 8.0. The sample was then buffer exchanged (Zeba spin column) into a hybridization buffer containing diethyl pyrocarbonate (DEPC)-treated 20 mM morpholinepropanesulfonic acid (MOPS) (pH 7.5), 329 mM NaCl, and 1 mM CaCl_2_ (MNC-Hy), 10% formamide, 10% polyethylene glycol (PEG) 6000, and 2 mM ribonucleoside vanadyl complex (RVC), after which 2.48 μl of 25 mg/ml yeast tRNA was added to each sample.

For experiments with unlabeled DLPs, a 50-fold molar excess of antibody 2B4 (Santa Cruz Biotechnology) was then added at room temperature and left for 15 min, followed by treatment with a 50-fold molar excess of an Alexa 488-labeled goat anti-mouse IgG (Thermo Fisher) for an additional 15 min.

For single-dye hybridization to transcripts produced by unlabeled and Atto 647N-labeled DLPs, Atto 565-labeled oligonucleotide probes were added to a final concentration of 270 nM. For two-dye oligonucleotide experiments, final concentrations of 932 nM Atto 647N- and 186 nM Atto 565-labeled oligonucleotides were added to samples containing Atto 488 labeled-DLPs. Hybridization was allowed to proceed at 37°C for 2 h, followed by desalting into MNC-Hy buffer and addition of 3 μl RNase inhibitor.

Five to 10 microliters of each sample was added to 500 μl MNC-Hy on a no. 1.5, 25-mm round coverslip (Warner Instruments), and particles were imaged as a single z-series over 15 z-planes with a step size of 0.35 μm. 2B4-coated DLPs were imaged at 488 nm at 50% power at 100 ms. Atto 488-labeled DLPs were imaged at 488 nm at 35% power at 100 ms. Atto 647N-labeled DLPs were imaged at 660 nm at 80% power at 100 ms. Oligonucleotide channels were imaged at full power with a 100- or 500-ms exposure times in the 561-nm channel and a 1,000-ms exposure in the 660-nm channel.

### *In situ* hybridization and immunohistochemical detection of NSP2 in rotavirus-infected cells.

BSC-1 cells were plated on round coverslips (previously sonicated for 20 min in 70% EtOH) in 6-well culture plates (Corning) and allowed to grow overnight to 50% confluence in supplemented DMEM. On the day of the experiment, the coverslips were washed twice with warmed MEM-α (Thermo Fisher Scientific) and infected with TLPs at an MOI of 20 in 2 ml MEM-α for 10 min at 37°C or mock infected with 2 ml of MEM-α. The cells were then washed twice in 2 ml of supplemented DMEM, and the infection was allowed to continue for a total of 6 h in supplemented DMEM.

After three washes with 2 ml of 20 mM HEPES (pH 7.5) 100 mM NaCl–1 mM CaCl_2_ (HNC), cells were fixed in 4% paraformaldehyde in HNC at room temperature for 10 min washed three times with HNC, permeabilized in HNC with 1% Triton X-100, again washed three times in HNC, and incubated for 1 h with 1 ml of anti-NSP2 antibody (MAb 32, a gift from John Patton, University of Maryland) diluted 1:500 in HNC plus 3% bovine serum albumin (BSA). After three more washes, samples were incubated with goat anti-mouse IgG labeled with Alexa 488 (Thermo Fisher Scientific) diluted 1:1,000 in HNC plus 3% BSA. After another round of washes, cells were incubated for 5 min in 2× saline sodium citrate (SSC), 30% formamide, and 2 mM RVC and then overnight at 37°C in 64 nM Atto 565-labeled oligonucleotide probe in hybridization buffer (12 μl per well) (2× SSC, 30% formamide, 2 mM RVC, 1 mg/ml yeast tRNA, 10% dextran sulfate); during this incubation, each coverslip was covered with a clean coverslip and the plate placed in a humidity chamber (plastic box with moist Kimwipe). The following day, all samples were washed three times in 2× SSC, postfixed in 4% paraformaldehyde in 2× SSC for 10 min, washed 3 times in SSC, and imaged as a single z-series, spanning the full cell volume with a step size of 0.35 μm, at 20% power with 20-ms exposure times in the 488 channel and at 50% power with 100-ms exposures in the 561-nm channel.

### Immunohistochemical detection of NSP2 in rcTLP-infected cells.

BSC-1 cells were plated and allowed to grow overnight as described above. On the day of the experiment, the coverslips were washed twice with warmed MEM-α (Thermo Fisher Scientific) and infected with rcTLPs (DLP = Atto 647N; VP7 = Atto 565) at an MOI of 1 in 2 ml MEM-α for 10 min at 37°C. The cells were then washed twice in 2 ml of supplemented DMEM, and the infection was allowed to continue for a total of 6 h in supplemented DMEM.

After three washes with 2 ml HNC, cells were fixed in 4% paraformaldehyde in HNC at room temperature for 10 min, washed three times with HNC, permeabilized in HNC with 1% Triton X-100, again washed three times in HNC, and incubated for 1 h with 1 ml of anti-NSP2 antibody diluted 1:500 in HNC plus 3% bovine serum albumin (BSA). After three more washes, samples were incubated with goat anti-mouse IgG labeled with Alexa 488 (Thermo Fisher Scientific) diluted 1:1,000 in HNC plus 3% BSA, followed by a final round of three washes. The samples were then imaged as a single z-series, spanning the full cell volume with a step size of 0.35 μm, at 20% power with 50-ms exposure times in the 488 channel, at 10% power with 100-ms exposures in the 562-nm channel, and at 90% power with 100-ms exposures in the 640-nm channel.

### *In situ* hybridization of cells infected with doubly labeled rcTLPs.

BSC-1 cells were plated and grown to 50% confluence as described above; MA104 cells were grown overnight to confluence in supplemented M199. After two washes with MEM-α (BSC-1) or M199 (MA104), doubly labeled rcTLPs were added to achieve the required MOI. For 5-h incubation experiments, rcTLPs (DLP-647N and VP7-488, MOI = 1 or 5) were allowed to bind and infect for 10 min before the cells were washed twice with MEM-α and the infection was allowed to proceed for a total of 5 h. For short-term infections, rcTLPs (DLP-647N and VP7-488, MOI = 20; DLP-488 and VP7-390, MOI = 5) were allowed to infect, without washing, for 15, 30, or 60 min. Mock-infected samples were treated similarly but without addition of virus particles.

At the end of the infection period, samples were washed three times in DEPC-treated 20 mM MOPS (pH 7.5), 100 mM NaCl, and 1 mM CaCl_2_ (MNC) and fixed in 4% paraformaldehyde in MNC at room temperature for 10 min. Three 5-min washes in MNC were followed by a quick rinse in MNC with 2 mM RVC (MNC-RVC) and a 5-min incubation in MNC-RVC with 0.1% Triton X-100. Three 5-min washes in MNC-RVC were then followed by a final incubation in DEPC-treated 20 mM MOPS (pH 7.5), 329 mM NaCl, and 1 mM CaCl_2_ (MNC-Hy) buffer with 10% formamide and 2 mM RVC.

For experiments with single-pool Atto 565-labeled oligonucleotide, a final concentration of 270 nM labeled probe was added to MNC-Hy buffer with 10% formamide, 10% polyethylene glycol (PEG) 6000, 2 mM RVC, and 1 mg/ml yeast tRNA. For dual-probe experiments, 27 nM Atto 565- and 135 nM Atto 647N-labeled oligonucleotide probes were added to the same buffer. Twelve microliters of this probe mixture was added to each coverslip, covered with a second clean coverslip, and incubated overnight at 37°C in a humidity chamber. The following day, all samples were washed three times in MNC-Hy with 10% formamide at 37°C for 10 min and stored in MNC at 4°C before imaging.

Images were collected as a single z-series spanning the full volume of the cells with a step size of 0.35 μm. For the single-probe pool, data were collected at 50% power with a 100-ms exposure time in the 488 channel, at 100% power with a 100-ms exposure time in the 561-nm channel, and at 80% power with a 100-ms exposure time in the 640-nm channel. Dual-probe data were collected at 70% power with a 100-ms exposure time in the 405-nm channel, at 35% power with a 100-ms exposure time in the 488-nm channel, at 100% power with a 100- or 500-ms exposure time in the 561-nm channel, and at 100% power with a 1,000-ms exposure time in the 640-nm channel.

### Confocal imaging.

All images were acquired with a Mariana system (Intelligent Imaging Innovations, Denver, CO) based on a Zeiss Axio-Observer inverted microscope (Carl Zeiss Microimaging, Inc., Thornwood, NY) equipped with a CSU-X1 spinning-disk confocal unit (Yokogawa Electric, Tokyo, Japan), a piezo-driven Z-translation, and linear encoded X&Y translations and controlled with SlideBook V5.0 (Intelligent Imaging Inc., Denver, CO). Excitation wavelengths were 405, 488, 561, and 640 nm (lasers were from Cobolt, Solna, Sweden); the emission filters were 452/45, 525/50, 607/36, and 680-nm long-pass (Semrock, Rochester, NY). Exposure times and laser settings for each experiment are outlined above.

### Image analysis.

The signals from single dyes were detected and quantified using custom MATLAB (MathWorks, Natick, MA) routines that fitted the amplitudes with a three-dimensional (3D) Gaussian fitting function (32). The anisotropic 3D Gaussian sigmas were determined experimentally from a measured point spread function and were fixed at 1.16/1.1 and 1.36/1.11 for *xy/z* in the 560-nm and 642-nm excitation channels, respectively. Single Atto 565 or Atto 647N dyes were imaged in 3D at 500-nm z-steps and subsequently bleached, at various exposure times, to generate linear regression standard curves (fitted amplitude versus exposure time). The single-molecule values at the imaged exposure were extrapolated, yielding fitted amplitudes of 105 ± 10 (mean ± standard deviation [SD] of the single-particle distribution) for a single Atto 565 at 100 ms and 525 ± 10 at 500 ms. Similarly, for Atto 647N, we obtained a single dye value of 1,261 ± 34 at a 1,000-ms exposure. These single-molecule and SD values were propagated to calculate the number of labeled probes colocalized with each DLP in the *in vitro* mRNA transcription assay and the colocalization of transcripts with DLPs in infected, fixed cells.

DLP and labeled oligonucleotide signals from *in vitro* transcription samples were detected using the MATLAB routines described above. Examination of all detections in the 640-nm (single oligonucleotide) or 488-nm (dual-oligonucleotide pool) channel resulted in thresholds below which a given signal was not considered a DLP and above which the detection was likely due either to aggregates or to spatially unresolved particles. For the Atto 647N-labeled DLPs, this threshold was set between an amplitude of 1,500 and 4,000 (Fig. 4D), while the threshold for Atto 488-labeled DLPs was set between 400 and 1,500 (Fig. 4G). A particle with a fluorescent signal that fell within these thresholds was scored as colocalized with oligonucleotide if the amplitude in the respective oligonucleotide channel was significantly higher than background (32).

DLP, VP7, and labeled oligonucleotide signals in fixed infected cells were also detected as described above, with thresholds for a valid, single DLP/TLP set between 900 and 4,000 for Atto-647N labeled particles (Fig. 7C) and between 200 and 750 for Atto 488-labeled particles (Fig. 8D). A particle was classified as a DLP if it did not have a significant colocalized VP7 signal; colocalization of oligonucleotide followed the same criteria as the *in vitro* assay analysis described above.

Mock infection colocalization data were generated using BSC-1 cells that had not been infected with labeled rcTLPs but were nevertheless probed with the corresponding Atto-labeled probe pools as described above. One hundred random positions were chosen per mock-infected cell probed at 60 min postinfection with the single Atto 565 oligonucleotide pool to represent an average of 100 “uncoating” events per cell (Table 2). Similarly, 40 and 30 random locations were chosen per cell for the dual-probe pool and MA104 experiments, respectively, reflecting the average number of uncoating events per cell seen in Tables 3 and 4 at 60 min postinfection. Atto 565 oligonucleotide signals at these random positions were quantified using the anisotropic 3D Gaussian fitting function described above. All positions were limited to above the coverslip and to the interior of the volume of the cells in question by using a 3-dimensional mask derived from the autofluorescence in the 488-nm channel of the mock-infected samples (32).
